# Improved Maturity and Ripeness Classifications of Magnifera Indica cv. Harumanis Mangoes through Sensor Fusion of an Electronic Nose and Acoustic Sensor

**DOI:** 10.3390/s120506023

**Published:** 2012-05-10

**Authors:** Ammar Zakaria, Ali Yeon Md Shakaff, Maz Jamilah Masnan, Fathinul Syahir Ahmad Saad, Abdul Hamid Adom, Mohd Noor Ahmad, Mahmad Nor Jaafar, Abu Hassan Abdullah, Latifah Munirah Kamarudin

**Affiliations:** Centre of Excellence for Advanced Sensor Technology (CEASTech), Universiti Malaysia Perlis (UniMAP), 01000, Kangar, Perlis, Malaysia; E-Mails: aliyeon@unimap.edu.my (A.Y.M.S.); mazjamilah@unimap.edu.my (M.J.M.); fathinul@unimap.edu.my (F.S.A.S.); abdhamid@unimap.edu.my (A.H.A.); mohdnoor@unimap.edu.my (M.N.A.); mahmad@unimap.edu.my (M.N.J.); abu.hassan@unimap.edu.my (A.H.A.); latifahmunirah@unimap.edu.my (L.M.K.)

**Keywords:** electronic nose, acoustic sensor, volatiles, mango ripeness classification

## Abstract

In recent years, there have been a number of reported studies on the use of non-destructive techniques to evaluate and determine mango maturity and ripeness levels. However, most of these reported works were conducted using single-modality sensing systems, either using an electronic nose, acoustics or other non-destructive measurements. This paper presents the work on the classification of mangoes (*Magnifera Indica* cv. Harumanis) maturity and ripeness levels using fusion of the data of an electronic nose and an acoustic sensor. Three groups of samples each from two different harvesting times (week 7 and week 8) were evaluated by the e-nose and then followed by the acoustic sensor. Principal Component Analysis (PCA) and Linear Discriminant Analysis (LDA) were able to discriminate the mango harvested at week 7 and week 8 based solely on the aroma and volatile gases released from the mangoes. However, when six different groups of different maturity and ripeness levels were combined in one classification analysis, both PCA and LDA were unable to discriminate the age difference of the Harumanis mangoes. Instead of six different groups, only four were observed using the LDA, while PCA showed only two distinct groups. By applying a low level data fusion technique on the e-nose and acoustic data, the classification for maturity and ripeness levels using LDA was improved. However, no significant improvement was observed using PCA with data fusion technique. Further work using a hybrid LDA-Competitive Learning Neural Network was performed to validate the fusion technique and classify the samples. It was found that the LDA-CLNN was also improved significantly when data fusion was applied.

## 1. Introduction

**Figure 1.**
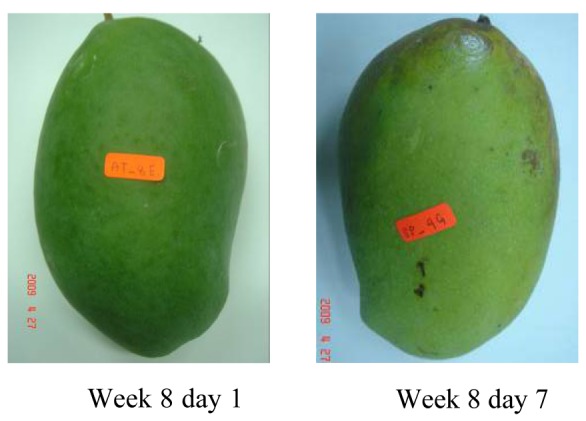
The skin colour of two different maturity stages, week 8 day 1 and week 8 day 7.

The perceived quality of mangoes is greatly dependent on their time of harvest and normally the quality is set according to their maturity stages. It has been widely known that there are many parameters can be used to determine maturity stages [1]. These include age, size, skin colour, firmness, and smell. Mango is a climacteric fruit, which means that its internal biochemical changes occur during respiration and it may still undergo further changes after it has been harvested. Volatile compounds, such as ethylene and aromatic hydrocarbons (terpene hydrocarbons) are released during the ripening process [2,3] and these contribute to the characteristic mango aroma. Generally, during maturity stages, the fruits experience a rapid burst in ethylene release, a sharp rise in carbon dioxide production and a decrease in oxygen levels [4,5]. This characteristic allows the possibility of predicting the optimal harvest date by looking at the odour patterns (often referred to as the ‘smellprint’) of the fruit's volatile compounds using an e-nose. In the case of Harumanis mangoes, the use of smell as a maturity indicator is a better option compared to visual and colour inspection. As illustrated in Figure 1, the skin colour of the Harumanis is not an appropriate parameter to determine the maturity and ripeness levels.

A number of successful studies on the use of e-nose to determine the maturity and ripeness stages of mangoes and several climacteric fruits have been reported [6–11]. In this paper however, the aim is to evaluate the robustness of maturity and ripeness classification using PCA and LDA. Two batches of Harumanis samples were harvested at week 7 (Green) and week 8 (Mature), and kept for 1 day at room temperature (27 °C) to homogenize before the measurements of the volatile compounds of the mangoes were taken on day 2, 4 and 7 after the harvest. When the volatiles data were classified using a global classification method, it is clear that the e-nose alone was insufficient to classify the small differences between maturity or ripeness levels of Harumanis mangoes. The classification method can be improved by adding more sensing parameters from different modalities.

The perceived quality of a mango also includes its texture and firmness. It is also one of the parameters in deciding whether the fruits have ripened and ready for consumption [12]. Firmness can be determined either destructively using a penetrometer or using non-destructive sensing such as acoustic sensors.

Furthermore, multi-modality sensor fusion was reported to give better classification of fruit maturity levels compared to a single modal system [13]. Natale [14] has successfully implemented a system to classify peaches based on the fusion of e-nose and visible optical spectroscopy data.

In this investigation, the classification of Harumanis mangoes maturity and ripeness levels were determined by combining e-nose and acoustic sensor data using a low level fusion technique. This combination is able to provide additional information and allows for better classifications of the maturity and ripeness levels of the mangoes.

This sensor fusion is performed by combining the information provided by different sensors in different modalities. It is introduced when data from two or more sensors are combined and mimicking the biological system. For example, sensor fusion of two different modalities such as e-nose and acoustics mimics the human sensory system in perceiving fruit maturity and ripeness levels. Similarly, fruit eating bats use a combination of odour-guided detection together with echolocation to distinguish the ripe fruits [15]. In essence, this technique allows the proposed system to mimic the consumer preferences in choosing the optimal quality of mangoes [16].

This paper describes the low level fusion of e-nose and acoustic data for the improved classification of different maturity and ripeness levels using PCA and LDA. Further classification was also performed using LDA-CLNN to validate the fusion technique and further classify the samples.

## 2. Materials and Methods

### 2.1. Sample Selection

In Perlis, Malaysia, Harumanis mangoes are usually harvested from the end of April until the middle of June. Typically, they are harvested between 60–120 d past flowering or about 8 weeks after the fruits reach the size of approximately 4 cm in diameter. Subsequently, the mangoes will be classified by trained personnel into six different categories based on physical size and appearance (as well as estimated maturity stages) as shown in Table 1. In this study, two batches were acquired from the Perlis State Department of Agriculture. Each batch contained fruits from two different harvesting dates [week 7 (Stage 0) and week 8 (Stage 1)] and each stage has 60 mango samples, thus in total, 240 mangoes were obtained for this experiment. Each stage was split into two different groups, whereby 14 mango samples (for each stage 0 and stage 1) were isolated and labelled as test samples and used for biochemical measurements using a destructive method. In total, 56 mango samples (for both batches) were used for the e-nose and acoustic measurements. The rest of the samples were used as a control sample for the e-nose and acoustic measurements. As the mango ripens, only the good samples were selected for the control measurements. The number of mango samples used for the e-nose measurement is described in Table 2 and each measurement was repeated five times. All the mango samples weighed 430 (±50) g each were examined carefully to ensure that they were free from physical and external damage.

### 2.2. Electronic Nose

The experiments were carried out using a Cyranose 320 e-nose from Smiths Detection (Pasadena, CA, USA). It has been used in many applications including quality control for the food industry, hazardous material identification, biomedical sample discrimination, plant disease detection and many others [17–21]. The main components of an e-nose include the odour capture module, sensing elements, data pre-processing and pattern recognition algorithms. The sensing elements consist of a 32 potentiometric sensor array made up of various conducting polymers, blended with carbon-black composite. These potentiometric sensors were designed to be partially selective. The combination of such sensors as an array introduces a cross sensitivity effect, which may even allow the discrimination and classification of complex volatile compounds [22,23]. The data collected and logged by the e-nose are the resistance values of the sensor array during contact with the volatile gases, which corresponds to the ‘smellprint’.

The e-nose has to be configured before it can be used. The main configuration parameter is the gas exposure. The sniffing process comprises of four different cycles: (i) baseline recovery, (ii) sample draw, (iii) idle time and (iv) purging. The flow rate of this sniffing process (baseline recovery, sample draw and purging) can be set at three different speeds (low: 50 mL/L, medium: 120 mL/L and high: 160 mL/L). The detail of the sniffing process is discussed in Section 2.5.1

### 2.3. Acoustic Firmness Measurements

The mango firmness was measured using an AFS unit (AWETA G&P, Nootdorp, The Netherlands). This device employs an acoustic technique that provides a non-destructive measurement. The acoustic signal was generated by a solenoid plunger that gently taps the fruits. The tapping (ticking) power that controls the plunger can be adjusted by the AFS V2.0 software. At the same time, a small microphone embedded in the flange of this unit captures the acoustic vibration waveform and the maximum peak of the ticking sound. Also, a small load cell was used to measure the weight of the mango.

This device has three main parameters: (i) microphone gain, (ii) ticking power and (iii) frequency range. Preliminary experiments were performed to obtain the optimal configuration setting. The most important parameters are the tick power versus microphone gain. The height of the scan in the ticking waveform must fit easily and must be ensured that the signal was not clipping. Table 3 shows the optimal configuration setting used in this experiment.

The AFS unit gives two different types of measurements. The firmness index (*FI*) is based on the acoustical measurement while the alternative firmness index (*AFI*) is based on the impact measurement that evaluates the local surface elasticity. The *FI* is defined as follows:

(1) $$FI={\text{M}}^{2/3}\times {{\text{f}}_{\text{o}}}^{2}/\text{scaling factor}$$

where f_o_ is the resonance frequency and M is mass of mangoes.

### 2.4. Sample Preparation

**Figure 2.**
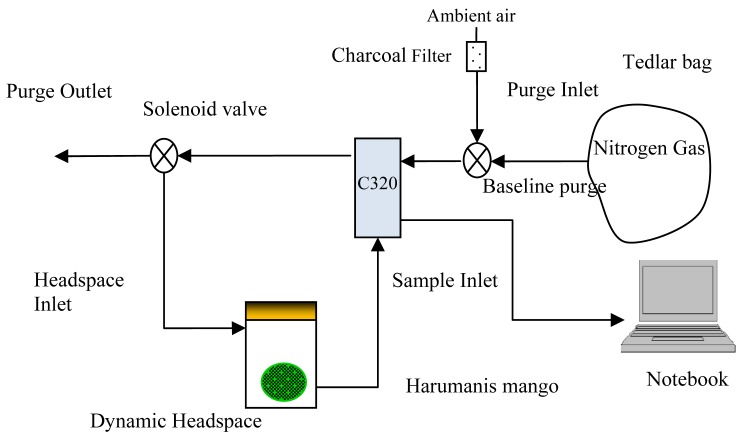
Block diagram of odour data collection setup.

Sample preparation is one of the most critical steps to ensure repeatable results. The volatile production might still vary with incubation time due to the small variations in the sample selection, such as weight, age and size. Thus, to obtain the optimal incubation time, three mango samples were picked from each of stage 0 and stage 1 for the initial experiment. Each sample was incubated inside the jar for 15 min and the odour measurements were repeated five times. Later, the same procedure was repeated at 30 min and 45 min of incubation time. Preliminary experiments showed that 30 min is the optimum headspace equilibrium, with the right amount of volatile composition to give strong discrimination between immature, mature, nearly ripe and ripe samples. At 45 min of incubation time, small water droplets were seen inside the surface of the jar and the e-nose measurements were affected by the humidity. On the other hand, at 15 min incubation time, the noise level was rather large (results not shown) and there is no distinct variation in the e-nose measurement of different mangoes samples. Prior to the odour measurements, the mango sample was sealed in an air-tight acrylic jar. The jar was then purged with dry nitrogen gas and left for 30 min at 26 °C (room temperature) until the headspace of the jar equilibrated. The dynamic odour measurement setup is shown in Figure 2.

### 2.5. Non Destructive Test (NDT)

#### 2.5.1. Odour Sampling

The e-nose has four main parameters for the odour sampling process. The sequence of the odour sampling started with a baseline recovery, followed by a sample draw, idle and finally purging process. During purging, the sensor chamber was flushed with ambient air (filtered by activated charcoal to remove volatile organic compounds (VOCs) in the background ambient), and later switched to nitrogen gas (which was filled in a tedlar bag) during the baseline recovery. This method is required to obtain a consistent reference baseline [24]. During the purging and baseline recovery, the gases were purge through the purge outlet. During the sniffing process, the gases were switched back to the jar to maintain the concentration of the headspace in the jar. The same method was adopted by [25] to minimize the concentration drift effect. Electro-mechanical solenoid valves were used and manually controlled to switch the direction of the purging gases and headspace gases.

Several sets of initial experiments were performed whereby the smellprint of 40 mango samples from two different maturity stages (Table 2) were compared over 15 s, 30 s and 50 s of sniffing time. The first readings were always discarded due to possible false readings from previous residual gases in the chamber and sampling system [25]. The purging time and speed was set higher (60 s with fast flow rate) to ensure the residual volatiles was adequately purged. The optimal purging duration can be validated by comparing the sensor response during baseline recovery. Excessive and longer purging should be avoided as it can cause temperature and temporal drift [26–28].

Upon obtaining the optimal setting, five sniffs were performed on each mango sample. Ten s were set as the baseline recovery (with medium flow rate); 50 s for sample draw (with medium flow rate); 3 s intermediate pause between sniffing and purging; and 60 s to finally purge the residue of previous sample gases (with fast flow rate). The e-nose configuration setting is shown in Table 4. Medium flow rate (120 mL/min) for odour sampling that gives the optimal response was adopted for this experimental setup. This is similar to several published work [29–32] using low analytes flow rates setup especially when dealing with conducting polymer sensor. The right choice of flow rates enabled the correct identification and quantification of aromatic volatiles released from the fruits. The odour measurement was performed on the mango samples in a random order. All online e-nose measurement data was saved in txt file format via serial cable and later extracted using MATLAB software.

#### 2.5.2. Acoustic Measurements

For the acoustic assessments, the mango firmness index (FI) and alternative firmness index (AFI) were measured at four different locations, such as top, bottom, right side and left side of Harumanis mango. Each measurement was repeated three times and the average was obtained.

### 2.6. Biochemical Measurements

#### 2.6.1. Brix and pH

The mangoes were analysed for total soluble solids (TSS), °Brix and pH level using digital refractometer (Reichert-AR200, Depew, NY, USA) and pH-meter (TESTO 206-pH2, Sparta, NJ, USA) respectively. Both measurements were set with automatic temperature correction. Each pH and Brix measurement was repeated at least three times and the average was obtained.

Each time after both the e-nose and acoustic measurements were taken, 14 mangoes randomly taken from the test samples were sliced from top to bottom. The juice from a mango was squeezed from the fruit and brix level was measured using refractometer. Similarly, the pH value was obtained using a testo-pH2 probe. Table 2 shows the number of samples that were used in this experiment. The destructive measurements of Brix and pH were used to validate and verify the two different groups of samples (green and mature) of three different dates by looking at the mangoes internal biochemical measurements.

#### 2.6.2. Gas Chromatography Mass Spectrometry (GC-MS)

For GC-MS analysis, Solid Phase Metal Extraction (SPME) needles made by CAR/PDMS (Supelco-57320-U, Bellefonte, PA, USA) was used to extract the headspace of the mango samples. Each sample was kept in an air-tight acrylic jar, purged with nitrogen and sealed (incubated) for at least 60 min. Subsequently, the SPME needle was exposed to the headspace of the mango for 30 min. The SPME needle was then inserted into the GC-MS inlet port for the analysis. The initial oven temperature was set to 40 °C for 1 minute, ramped 10 °C/min to 200 °C and held for 5 min. Nitrogen was used as the carrier gas. Nine samples were used for this analysis: Week 8 day 2 (3 samples), Week 8 day 4 (3 samples), and Week 8 day 7 (3 samples). The headspace compound was analysed using Perkin Elmer Clarus 600 Gas Chromatography (Clarus 600 T Mass Spectrometer; Turbomass Software 5.4.2, USA). The headspace compound identification was done by looking at the retention time and comparing with the known library standard.

### 2.7. Pre-Processing of E-Nose and Acoustic Data

Before the analysis, fractional measurement, *S_frac_* was applied to pre-process the raw e-nose data. This is often known as baseline manipulation. The baseline is subtracted and then divided by the sensor response. The result is a dimensionless and normalized *S_frac_*, where:

(2) $$S_{\text{frac}}=[{\text{S}}_{\max}-{\text{S}}_{0}]/{\text{S}}_{0}$$

This baseline manipulation generates a unit response for each sensor array output with respect to the baseline, which compensates for sensors that have intrinsically large varying response levels. It can also further minimize the effects of temperature, humidity and temporal drifts. S_0_ (baseline reading) is the reading of the reference gas (nitrogen), while S_max_ is the maximum sensor readings when measuring the headspace of Harumanis samples. The sniffing cycle was repeated 5 times for each mango sample. After the above operation, the data *S_frac_* was further scaled to zero mean and one standard deviation. This is to ensure that all sensor responses were standardized and no particular sensor dominates the result.

For the acoustic measurements, the average values of FI and AFI for each sample were obtained. These measurements were also scaled to zero mean and one standard deviation. Data from different modalities were processed separately and scaled before being fused together.

An *exploratory* data analysis technique, in this case PCA, was identified as a suitable method to visualize patterns in the data. Each individual modality was projected separately by PCA. An adequate number of dimensions projected by PCA were determined based on principal components (PCs) that have achieved cumulative variance of 80% or more. Further trial to classify and validate the samples using a classification method was performed using LDA. While, LDA is a supervised pattern classification method and is based on the determination of linear discriminant functions of which inter-group variance is maximized and within-group variance is minimized. Cross-validation using leave-one-out method was performed using LDA and none of the training samples were used for the testing process. The PCA and LDA were computed using MATLAB 7.0 and SPSS 17.0, respectively.

### 2.8. Classification Using Artificial Neural Network (ANN)

**Figure 3.**
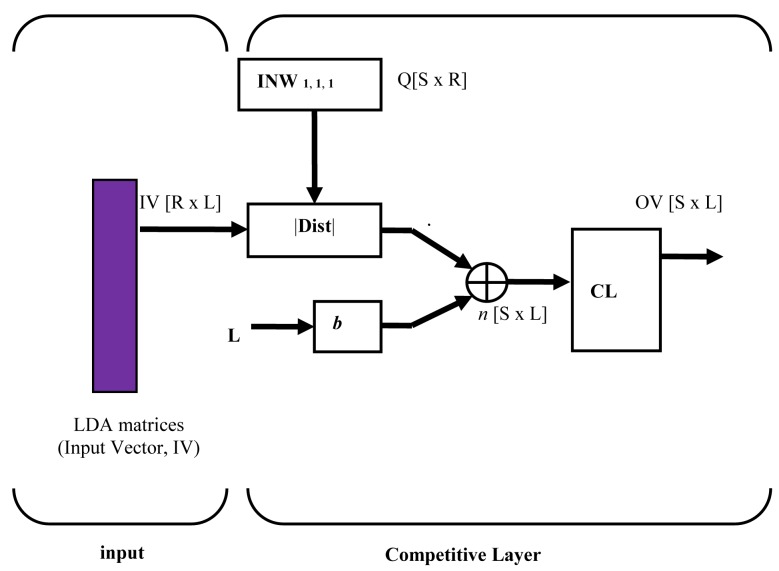
The architecture of LDA-CLNN [35].

Generally, ANN is a powerful method that can be used to classify and predict unknown samples. ANN can be divided into supervised and unsupervised techniques. In most cases, supervised ANN such as Multilayer Perceptrons (MLP), Backpropagation (BP) and Radial Basis Function (RBF) are non-graphical and require higher processing power, while unsupervised ANN such as Hopfield Network (HNN), Competitive Learning (CLNN) and Self-Organizing Maps (SOM) produce a graphical representation and the computations are less complex [33–35]. Another advantage of unsupervised neural networks is that they are able to provide online classification. In this case, further validation and analysis to classify the six different classes from the LDA were performed using a hybrid graphical ANN known as LDA-CLNN [34]. The input vectors were taken from the first three components of the discriminant score of LDA output and fed through CLNN competitive layer. The size of input vector can be more than two vector matrices. The input and architecture layer for a LDA-CLNN is shown in Figure 3. The CLNN architecture is quite similar and related to Hamming network. The neurons in this competitive layer distribute themselves to recognize the behaviour of the input vectors taken from the discriminant score of LDA output.

The parameter |**Dist**| in Figure 3 was formed by the distances between the input vector, ***p*** and input weight matrix, **IW**. The **Dist** matrix has positive elements [**SxL**] which are later added with biases, ***b*** to compute the net input ***n*** of a competitive layer. If all biases are zero, the maximum net input a neuron can have is ‘0’. This occurs when the input vector ***p*** equals the neuron's weight vector. The competitive transfer function **CL**, accepts a net input vector **n**^1^ for a layer and returns neuron outputs of ‘0’ for all neurons except for one neuron (the *winner*). The winner neuron is associated with the highest element of net input **n**^1^.

**Figure 4.**
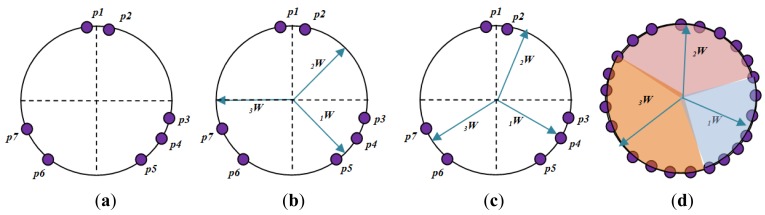
Graphical representation of competitive layer learns to classify input vectors.

Figure 4 shows how a competitive layer learns to classify seven input vector with 3 neurons. The seven input vectors illustrated in Figure 4(a) are as follows:

$$p1=\left[\begin{array}{c} -0.198\\ 0.974 \end{array}\right],p2=\left[\begin{array}{c} 0.196\\ 0.981 \end{array}\right],p3=\left[\begin{array}{c} 0.988\\ -0.214 \end{array}\right],p4=\left[\begin{array}{c} 0.824\\ -0.512 \end{array}\right],p5=\left[\begin{array}{c} 0.524\\ -0.812 \end{array}\right],p6=\left[\begin{array}{c} -0.351\\ -0.875 \end{array}\right],p7=\left[\begin{array}{c} -0.725\\ -0.625 \end{array}\right]$$

The competitive network is set up with three neurons, which represent the target number of sample classes. Thus, it can classify vectors into three classes only. The arrows in Figure 4(b) represent “randomly” chosen initial and normalized weights. The three random weights are shown as follows:

w1=[0.707−0.707],w2=[0.7070.707],w3=[−1.0000],W=[W1TW2TW3T]

When ***p**2* is presented to the CLNN network, the second neuron's weight vector was the closest. So it won the competition and the output a 1. Then Kohonen learning rule was applied to the winning neuron with a learning rate of *α* = 0.5.

W2new=W2old+α(p2−W2old)=[0.7070.707]+0.5([0.1960.981]−[0.7070.707])=[0.4510.844]

The *Kohonen* rule moves _2_w closer to ***p**2*, as illustrated in Figure 4(c). As the input vector keeps on presenting to the network, then at each iteration, the weight vector that is closest to the input vector will move towards that vector. Eventually, each weight vector will point at a different cluster of input vectors. Each weight vector becomes a model for a different cluster. Once the network has learned to cluster the input vectors, it will classify new vectors accordingly as shown in Figure 4(d).

In summary, the competitive layer assigns each input vector **p** to one of those three classes by producing an output of 1 for the neuron whose weight vector is closest to **p**.

The Bias learning rules in LDA-CLNN were adopted for the training of the network. Unlike other ANN, LDA-CLNN can be understood better when their weight vectors and input vectors are shown graphically. The training for both the e-nose measurement and the combined e-nose with acoustic (data fusion technique) was performed separately with 200 epochs and repeated three times. Since there are six different groups, the number of neurons is set to six to classify the input vectors into six different groups.

### 2.9. Data Fusion

Low level fusion is performed by combining the information provided by different sensors of different modalities. There are many methods to perform this fusion *i.e.*, using neural networks [36,37], template methods and cluster algorithms. In this experiment, PCA, LDA and LDA-CLNN were chosen to perform the low level fusion. The requirement for this method is that the sensors for both modalities must commensurate.

PCA was used to analyze the behaviour or the grouping of the data. Further training, validation and classification between sample groups of the data fusion were performed using LDA. Cross-validation using leave-one-out method was carried out and variable selection was accomplished using Wilks' lambda test. Fisher linear discriminant function was also applied in this analysis.

Both the e-nose and acoustic data consists of 480 samples with 32 variables and two variables, respectively. Hence, the combined dataset from the e-nose and acoustic consists of 480 data samples with 34 variables. To ensure these datasets are standardized, the new dataset (after being combined) was scaled before performing the PCA, and LDA.

## 3. Results and Discussion

### 3.1. Biochemical Measurement Results

After being harvested, 14 mango samples (seven mangoes taken each from week 7 and week 8) were sliced from top to bottom. The pH and brix measurement were obtained and repeated three times. The measurement was repeated on the second batch of samples and the summary of pH and brix levels is given in Table 5. Further measurements of pH and brix were carried out throughout day 2, 4 and 7 as shown in Table 5. The result shows that mangoes harvested early are less sweet and slightly sour compared to the mangoes harvested at week 8. The same results were reported by [38,39].

**Figure 5.**
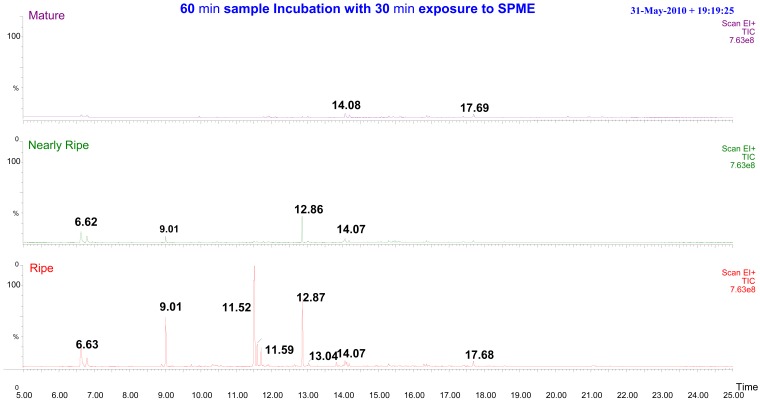
Three different maturity and ripeness levels of Harumanis mango headspace extraction using SPME.

Further work using GC-MS was also performed to investigate the volatile compounds emitted from the mangoes. This conventional method using SPME to extract the headspace of Harumanis mangoes for GC-MS analysis requires at least 60 min and an additional 30 min of SPME exposure time to the mango headspace. This lengthy process is required to obtain consistent and repeatable readings. Shorter exposure time of SPME to the headspace resulted in fewer peaks being observed in the chromatography plot. The GC-MS analysis result of 60 min incubation and 30 min SPME exposure time is shown in Figure 5. The variation of headspace volatile compounds was observed from an intact mango at three different maturity and ripeness levels *i.e.*, mature, nearly ripe and ripe. The compositions of the 12 most prominent volatile compounds are shown in Table 6. The peak was compared and matched with the known NIST and Wiley mass spectra library. Each ‘peak search’ produces a “hit list” by the spectra library, which is ordered by similarity to the target spectrum according to a computed “match factor”. Ideally, the match factor should reflect the likelihood that the search peak and reference spectrum arose from the same compound. The distinct variation of headspace volatiles for the different ripeness levels strengthens the idea of using e-nose as a non-destructive tool to evaluate mango maturity and ripeness.

### 3.2. FI and AFI Measurement Results

All mango samples were kept in a room with constant temperature of 27(±1) °C and humidity of 80% during the experimentation process. The FI and AFI were also measured from the control samples at two different harvesting dates (week 7 and week 8). Seven random samples were taken from the test samples for brix and pH measurements. The same method and experiments were repeated for the second batch of samples. Table 7 show both the firmness index and alternative firmness index, respectively. The FI in Table 7 shows mangoes that were harvested at week 7 were firmer compared to the mango samples that were harvested at week 8. However, after seven days, the measurement shows that the control samples harvested at week 7 shrivel faster than the control sample harvested at week 8. The ripening process or degrading (rotting process) started earlier in mangoes harvested during their premature stages [1,38,39].

**Figure 6.**
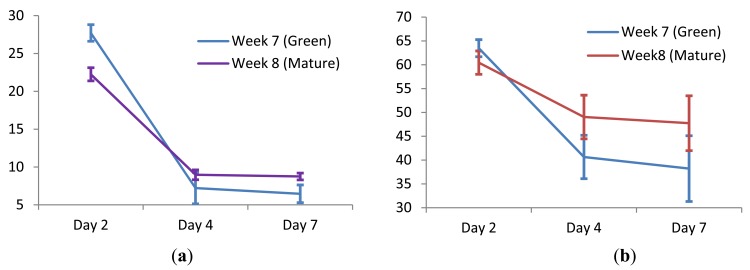
(**a**) Firmness Index (FI) and (**b**) Alternative Firmness Index (AFI) plot of mangoes harvested at Week 7 (Green) and Week 8 (Mature) on three different days

Figure 6 shows that there are strong correlations of FI and AFI after 2, 4 and 7 days of two different groups (green and mature) samples. However, the FI and AFI are less accurate without the prior information on the date of harvesting and duration of shelf life. Thus, another sensor modality such as e-nose can be coupled with FI and AFI to give better classification results.

### 3.3. E-Nose Measurement Results

Prior to performing e-nose measurements, the optimal configuration parameters must be obtained. Several experiments were performed to determine the best possible experimental condition. Odour sensor chamber was set to 33(±1) °C to avoid temperature drifting or fluctuation that might affect the measurement. The acrylic jar was purge with dry nitrogen gas before starting to incubate the mango sample for 30 min. This procedure is important to avoid contamination from ambient air and humidity. This strict procedure is to ensure higher repeatability and reproducibility during the measurements.

**Figure 7.**
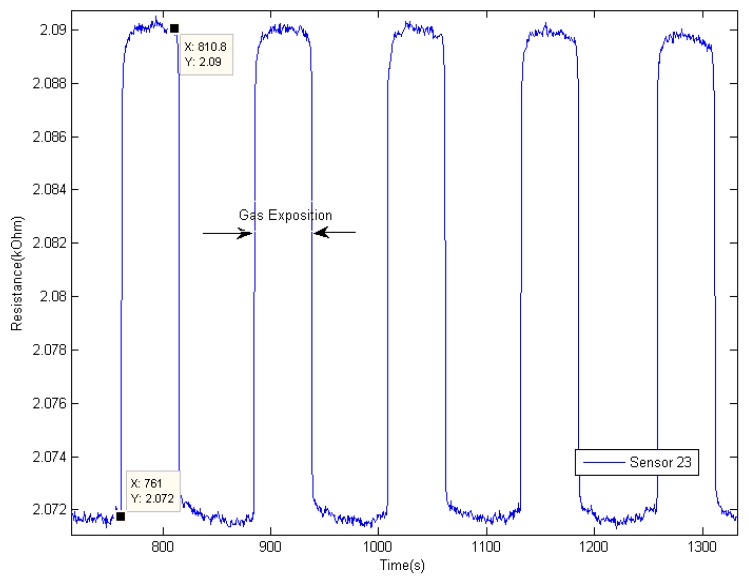
A complete cycle of six repeated measurements on mango sample taken at week 8.

Figure 7 shows high consistencies between repeated measurements. The sensor has reached steady-state response when sniffing was performed for about 50 s. The performance may be better with longer sniffing time. However, there is a drawback when performing longer sniffing time. The headspace will be depleted faster and will result in huge variation on the 4th and 5th of repeated measurement. Temperature and temporal drifts might affect the result as the measurement process will take longer than usual. It may also require longer purging time to ensure the previous residual volatile is totally removed from the conducting polymer sensor.

**Figure 8.**
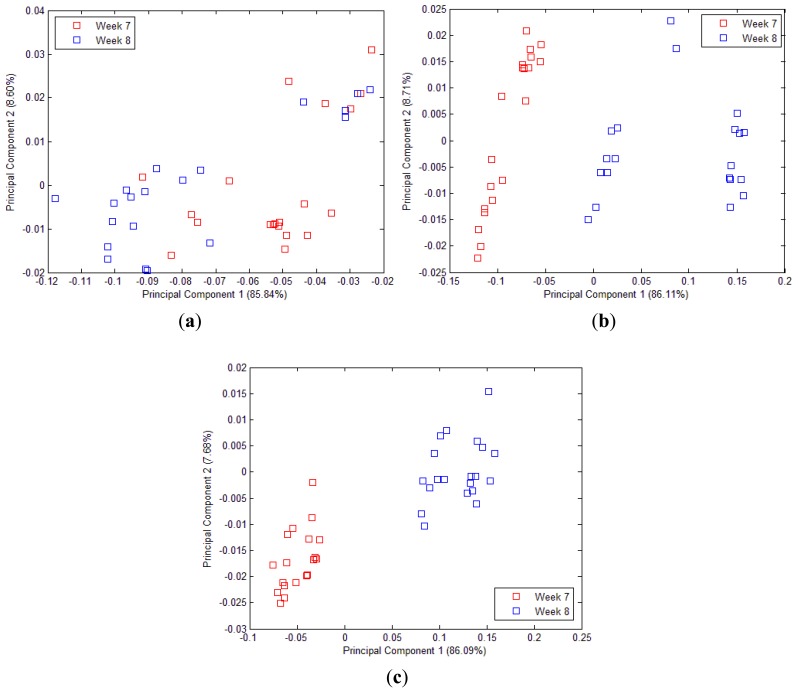
(**a**) PCA plot of 40 mango samples harvested at week 7 and week 8 with 15 s sniffing time. (**b**) PCA plot of 40 mango sample harvested at week 7 and week 8 with 30 s sniffing time. (**c**) PCA plot of 40 mango samples harvested at week 7 and week 8 with 50 s sniffing time.

The projected PCA in Figure 8(a–c) shows the fingerprint (‘smellprint’) of volatile assessment of 40 mango samples [20 samples each from week 7 (Green) and week 8 (Mature)] for 15 s, 30 s and 50 s sample draw, respectively. Two distinct groupings with higher repeatability and reproducibility were observed in the PCA projection in Figure 8(c) while the inconsistent grouping was observed in Figure 8(a,b). The projected PCA in Figure 8(c) shows the sensor correlation (which is linearly separable) between two different maturity level that were harvested at week 7 and week 8. Thus, 50 s of sample draw is the optimum setup to differentiate mangoes harvested at week 7 and week 8.

The optimal configuration setup was implemented throughout the experiment for the e-nose measurement. The volatile compounds from the dynamic headspace of each mango (explained in Table 2) was measured and repeated three times. The measurements were performed on day 2, day 4 and day 7 after harvesting.

**Figure 9.**
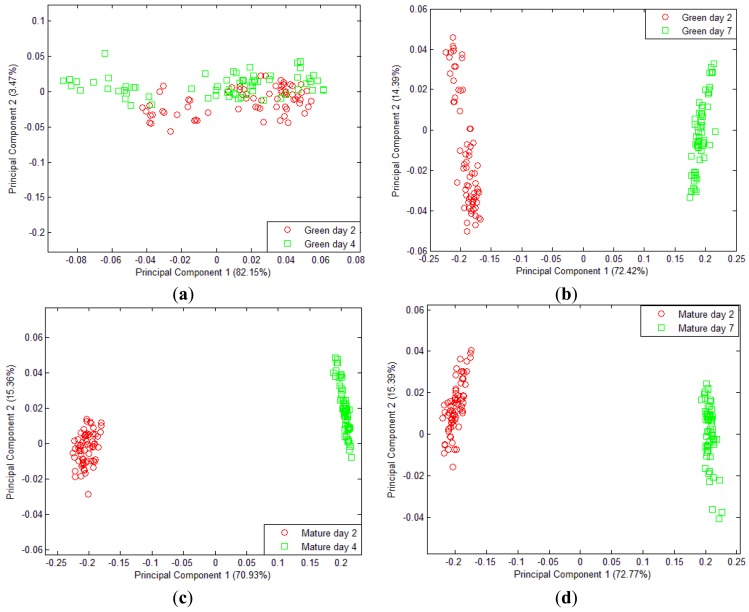
PCA plot of 32 mango sample (**a**) harvested at week 7 measured on day 2 and day 4, (**b**) harvested at week 7 measured on day 2 and day 7, (**c**) harvested at week 8 and measured on day 2 and day 4 and (**d**) harvested at week 8 and measured on day 2 and day 7.

All mango samples that were harvested on week 7 and week 8 were labelled as Green and Mature, respectively. Figure 9(a) shows that there is no distinctive difference in aroma detected by the e-nose between Green day 2 and Green day 4. However, the distribution of Green day 4 data is spread and can be actually split further into two different groupings. For Green day 7, the volatiles emission starts to differ. Figure 9(b) shows that there is clear separation between Green after 2 days and after 7 days. This is due to the amount of volatiles released and the presence of aromatic gases such as monoterpenes, sesquiterpenes, esters and other volatiles gases as the mangoes are now moving toward in the ripening process as shown and explained in Figure 5 and Table 6. A similar result was also reported by Lalel [40].

Interestingly, the volatile emissions from Mature day 2 and day 4 after harvest at week 8 show a quite distinct separation. During this period, between day 2 and day 4, the volatile gases are very distinguishable. At this time, there is a sharp rise in the amount of ethylene released by the mangoes [5]. However, in Figure 9(c), the separation between Mature day 2 and day 4 of the mature sample is more or less similar to the result in the Figure 9(d). The same result was observed as the mango starts ripening; most of the volatiles and aromatic gases released from the fruit begin to saturate [40].

**Figure 10.**
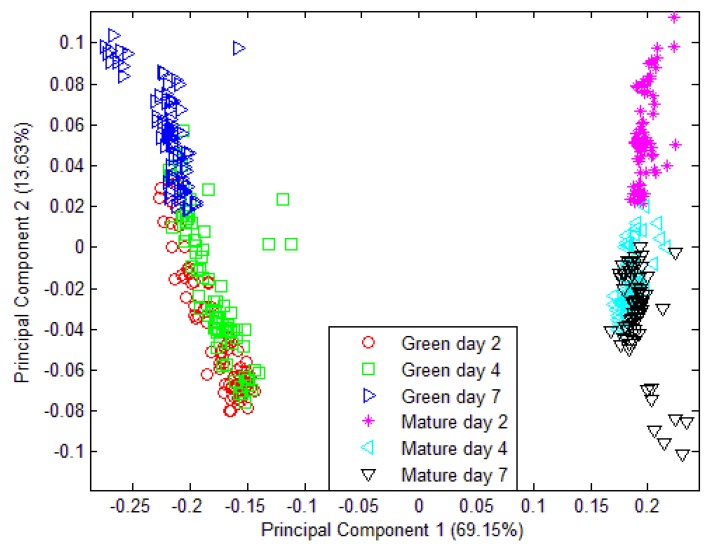
PCA for three different maturity and ripeness level of Harumanis harvested at two different dates Green (week 7) and Mature (week 8).

**Figure 11.**
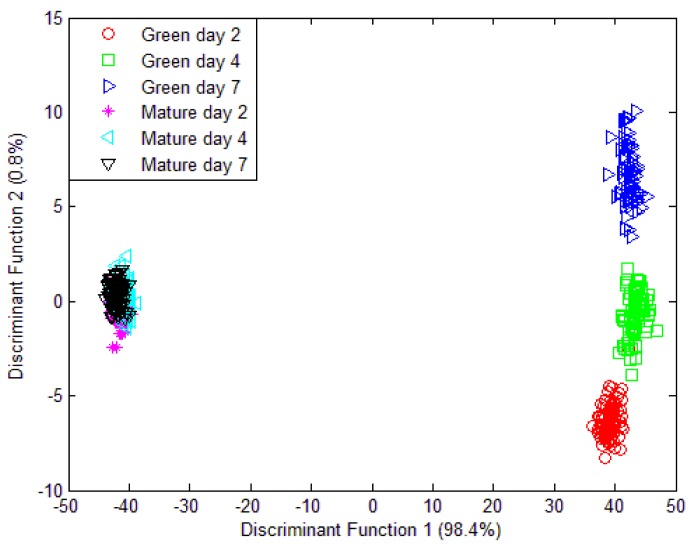
LDA plot of 32 e-nose sensors responses for three different maturity and ripeness level of Harumanis harvest at two different date (week 7 and week 8).

Unfortunately, when a classification model was implemented which consist of volatiles from mango samples at day 2, day 4 and day 7 that were harvested at week 7 and week 8; the PCA model was unable to discriminate the six different groupings. Only two different groupings were observed in Figure 10 and all 32 sensors were used in this model. Further validation and classification were performed using LDA. Similarly, the LDA plot observed in Figure 11 is unable to classify six different groups. The LDA can only discriminate four groupings instead of six different groups.

It can be observed that e-nose can further classify mangoes of day 2, 4 and 7 that were harvested at week 7 (green) compared to those samples that were harvested in week 8 (mature). E-nose is still unable provide better classification on mangoes samples that were harvested at week 8 although they are releasing distinctive aromatic volatiles between day 2, 4 and 7 as shown in Figure 5. The masking effects and synergism between different aromatic volatile compounds is observed in Figure 11 when mango samples (harvested at week 8) emits several distinctive aromatic volatiles but still grouped under one cluster. Similar finding was published by Hallier [41] on odour characterization of cooked Silurus *glanis*. This shows that e-nose system alone is insufficient to provide accurate classification compared to GC-MS system. This finding lead toward the fusion of e-nose and acoustic system to form a multi modalities sensing technique that holistically mimic the way human panel assess the fruit maturity and ripeness level.

### 3.4. Fusion of E-Nose and Acoustic Sensor

**Figure 12.**
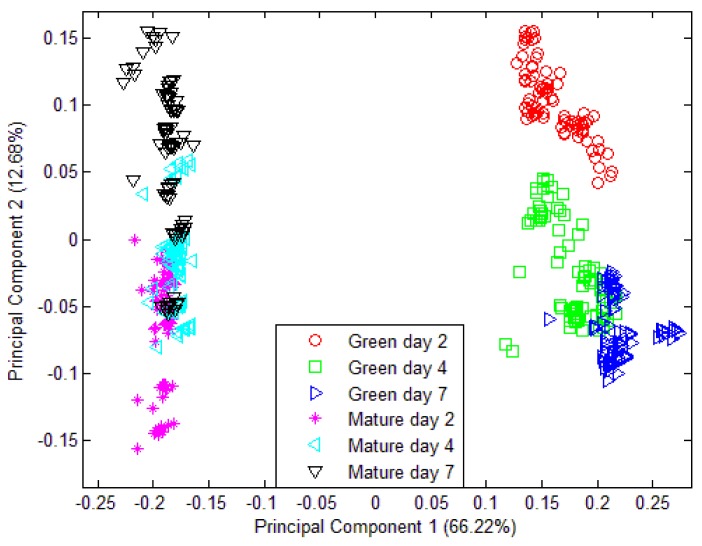
PCA plot using data fusion technique for three different maturity and ripeness level of Harumanis harvest at two different date (week 7 and week 8).

The limitation of PCA and LDA to produce a classification model of maturity and ripening level using e-nose measurement alone justifies the idea of introducing a fusion technique. The low level fusion technique was implemented using PCA and LDA. The PCA shows a slightly better grouping and separation as compared to the previous. The PCA plot of fused e-nose and acoustic measurement is shown in Figure 12. However, the result of this fusion method using PCA is still not satisfactory.

**Figure 13.**
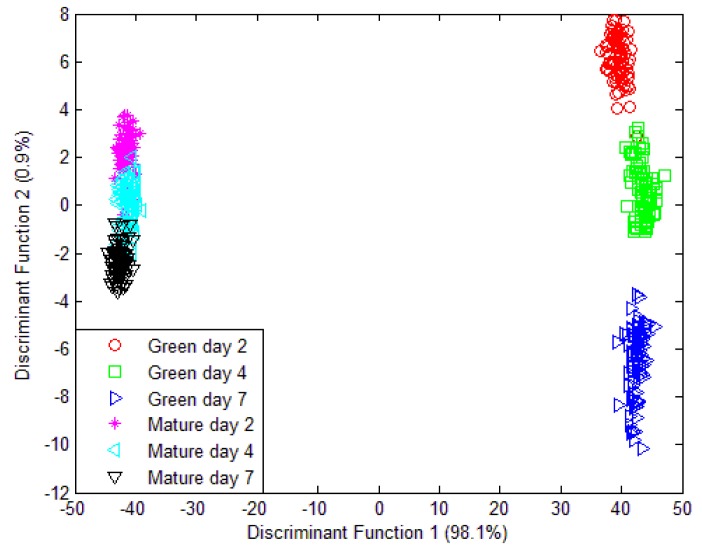
LDA plot using data fusion technique for three different maturity and ripeness level of Harumanis harvest at two different date (week 7 and week 8).

On the other hand, LDA shows a better performance. The LDA plot of data fusion technique in Figure 13 clearly shows the separation of six different groupings. The LDA model was tested and 100.0% of original grouped cases correctly classified and 98.8% of cross-validated grouped cases correctly classified. The classification result is shown in Table 8. The LDA was expected to give better classification. This is because PCA is one of the unsupervised exploratory data analysis techniques that convert a set of observations of correlated features into a set of values of uncorrelated variables that give meaningful graphical representation or the abstraction of the data. It is often used to reveal the behaviour of the data in a way which best explains the variance in the data. LDA, on the other hand, is a supervised pattern classification where the main objective is to maximize class discrimination and minimized within group variance. The objective of PCA is to squeeze variance into as few components as possible.

Further improvement toward this model can be made by selecting the more effective sensors. The sensor selection was performed using Wilks' lamda test shown in Table 9. Sensors 14 and 17 from the e-nose were found to be insignificant and can be dropped from the model.

### 3.5. ANN Results

Further validation and classification of Harumanis maturity and ripeness level was performed using LDA-CLNN. During training, each neuron in the layer closest to a group of input vectors adjusts its weight vector towards those input vectors. Eventually, if there are enough neurons, every cluster of similar input vectors has a neuron that outputs “1” when a vector in the cluster is presented, while outputting a “0” at all other times. Thus, the competitive network learns to categorize the input.

**Figure 14.**
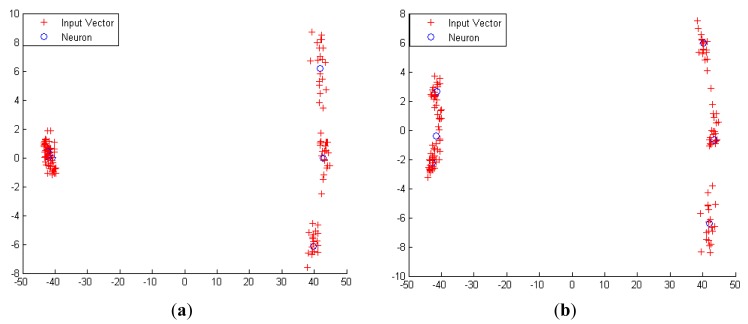
The graphical representation of LDA-CLNN with (**a**) 6 neurons of e-nose data (**b**) 6 neurons of fused e-nose and acoustic data on six different maturity and ripeness level.

The diagram in Figure 14 shows 480 three-element LDA input vectors represented with “+” markers. The competitive layer is set to have six neurons to classify the input vectors into six different groups as shown in Figure 14(a,b). These three-element input vectors (from LDA output) are adequate enough for the LDA-CLNN classifier to perform the prediction with 84.4% prediction accuracy. Vast improvement can be seen when the classifier is trained with the fused data rather than using only the e-nose data. The prediction accuracy was improved from 66.7% to 84.4% as shown in Table 10. Six different groups were successfully classified when the fused data was used compared to e-nose data where only four grouping were classified.

## 4. Conclusions

The GC-MS and e-nose results have shown that mango samples from two different harvesting times at Green (week 7) and Mature (week 8) produced very distinct aromatic smells and volatile gases. The firmness was also distinct between Green and Mature sample. For such cases, the PCA and LDA were adequate enough to discriminate and classify the samples according to different harvesting time. Similar results were also observed from FI and AFI.

However, when a classification model was performed based on e-nose data of six different groups, PCA and LDA were unable to separate the samples from week 7 and week 8 after 2, 4 and 7 days. Using PCA, the e-nose was able to discriminate only two out of six different groups. While for the LDA model, only four different groups were observed.

Furthermore, the use of FI and AFI solely to discriminate maturity and ripeness level of mangoes of unknown harvesting date are not very effective. A more precise time of harvest must be obtained for FI and AFI to work efficiently. Unfortunately, the exact date for harvesting is hard to achieve based on the physical parameters such as counting the age of the fruit from the day the flowers set, firmness or the skin colour.

By applying low level data fusion technique, the discrimination and classification of maturity and ripeness level using LDA was greatly improved. However, low level fusion using PCA was not satisfactory. The use of a low-level data fusion technique with the LDA for the e-nose and acoustic has enabled the six different groups of Harumanis mangoes to be grouped separately. These six groupings could also be associated with human preferences as it conveys the internal biochemistry and external parameter of mango characteristic. Moreover, this fusion technique has also improved the confidence level and discrimination performance by reducing uncertainties and allowing the e-nose and acoustic sensor to complement each other. Thus, this technique can extend the ability of e-nose and acoustic when fused together to evaluate and classify the maturity and ripening stages of the mango samples.

This investigation has proven that different sensor modalities can provide different and complementary information and hence by combining the modalities, the classification performance can be enhanced. This approach has enabled the evaluation and extraction of more information out of mango samples which have high similarities between them.

In summary, by applying data fusion, the combined e-nose and acoustic responses essentially mimicsed the human preference as both interact and complement each other. Hence, this fusion method has strong potential to assist human panels in making decisions, for applications such as fruit quality assessments. More modalities can be added in the near future such as NIR spectroscopy or IR vision to provide additional parameters for a more robust quality assessment of Harumanis mangoes.

## Figures and Tables

**Table 1.** Description of maturity stages and ripeness level of Harumanis mangoes.

**Stage**	**Age**	**Description**
0	Week 0–7	Pre-mature; Not yet harvested.
1	Week 8 (day 1)	Mature; Can start harvesting.
2	Week 8 (day 2)	Mature; Right after being harvested, cleaned, warm water treatments, and sorted based on weight and skin cleanness.
3	Week 8 (day 6)	Early ripening; Artificial ripening with calcium carbide. Smooth skin, and slightly bleak.
4	Week 8 (day 7)	Nearly ripe; Smooth, slightly soft skin and having slightly sweet aromas.
5	Week 9 (day 1)	Optimum ripeness; Smooth skin, soft and strong sweet aromas.

**Table 2.** Number of samples used in the e-nose, AFS measurement and biochemical measurements.

**Sample**	**Batch**	**Week 7**			**Week 8**		
		**Day 2**	**Day 4**	**Day 7**	**Day 2**	**Day 4**	**Day 7**
Control Sample ^a^	1	16	16	10	16	16	11
	2	16	16	8	16	16	10
Test Sample ^b^	1	14	14	14	14	14	14
	2	14	14	14	14	14	14

a The same mangoes were used in day 2, 4 and 7 for non-destructive e-nose and acoustic measurement.

b Destructive test and the mangoes were discarded after the pH and Brix measurements.

**Table 3.** Optimal setting configuration for AFS.

Parameters	Value
Measurements	3
Average Method	Mean
Microphone gain	87
Tick power	13.0
Frequency	50–1,950 kHz
Alternative firmness,	Impact
Min Valid Impact	1
Max Valid Impact	500
Min Valid Firmness	1
Max Valid Firmness	100

**Table 4.** E-nose parameter setting for Harumanis mango.

**Cycle**	**Time(s)**	**Pump Speed**
Baseline Purge	10	120 mL/min
Sample Draw	50	120 mL/min
Idle Time	3	–
Air Intake Purge	60	160 mL/min

**Table 5.** **(a)** pH and Brix levels for four different maturity and ripeness levels of Harumanis; **(b)** Brix and pH for four different maturity and ripeness levels that were harvested on two different dates.

**(a)**		

**Harvest date** ^a^	**pH**	**Brix**
Week 7 (Green)	3.18	17.10
Week 8 (Mature)	4.22	17.39

**(b)**						

**Harvesting time** ^b^	**Day 2** ^c^		**Day 4** ^c^		**Day 7** ^c^	

	**Brix**	**pH**	**Brix**	**pH**	**Brix**	**pH**
Week 7 (Green)	18.9	4.93	20.3	5.37	21.6	5.54
Week 8 (Mature)	20.6	4.93	21.3	5.99	24.4	6.22

a Week after the mango is about 4 cm in diameter.

b The mangoes were harvested at week 8 and week 7 after the size is about 4 cm and measured 2 days, 4 days and 7 days after.

c The average readings of three repeated measurements.

**Table 6.** Volatile compounds emitted from ripe, nearly ripe and ripe Harumanis mangoes.

**Peak**	**Retention Time**	**Compound Name**	**Mature**	**Nearly Ripe**	**Ripe**	**Match**
1	2.083	Ethylene	+	+	+	578
2	6.627	*α*-Pinene	+	+	+	864
3	6.789	*β*-Pinene	+	+	+	858
4	9.014	Octanoic acid, octyl ester	-	+	+	892
5	10.342	Pentyl Octanoate	-	-	+	749
6	11.516	1-Dodecen-3-yne	-	+	+	746
7	11.593	4-Decenoic acid, ethyl ester, (Z)-	-	+	+	840
8	11.701	Decanoic acid, ethyl ester		+	+	879
9	12.867	Cedrene	-	+	+	825
10	13.044	1,5-Cyclodecadiene, 1,5-dimethyl-8-(1-methylethylidene)-,(E,E)-	-	-	+	850
11	14.070	Diethyl Phthalate	+	+	+	798
12	17.683	Hexadecanoic acid, methyl ester	+	+	+	852

**Table 7.** FI and AFI for four different maturity and ripeness levels of Harumanis harvested on two different dates.

**Harvesting time** ^a^	**Day 2** ^b^		**Day 4** ^b^		**Day 7** ^b^	
	**FI**	**AFI**	**FI**	**AFI**	**FI**	**AFI**
Week 7 (Green)	27.71	63.48	7.213	40.64	6.463	38.21
Week 8 (Mature)	22.25	60.44	8.975	49.04	8.751	47.73

a The mango were harvested at week 8 and week 7 after the diameter is about 4 cm and measured 2 days, 4 days and 7 days later.

b The average readings of three repeated measurements.

**Table 8.** Classification results ^a^^,^^b^ on the fused e-nose and acoustic sensor using LDA for three different maturity and ripeness levels of Harumanis harvested on two different dates (week 7 and week 8).

		**Group**	**Predicted Group Membership**						**Total**
			**1.00**	**2.00**	**3.00**	**4.00**	**5.00**	**6.00**	
Original	Count	1.00	80	0	0	0	0	0	80
		2.00	0	80	0	0	0	0	80
		3.00	0	0	80	0	0	0	80
		4.00	0	0	0	80	0	0	80
		5.00	0	0	0	0	80	0	80
		6.00	0	0	0	0	0	80	80
	%	1.00	100.0	0.0	0.0	0.0	0.0	0.0	100.0
		2.00	0.0	100.0	0.0	0.0	0.0	0.0	100.0
		3.00	0.0	0.0	100.0	0.0	0.0	0.0	100.0
		4.00	0.0	0.0	0.0	100.0	0.0	0.0	100.0
		5.00	0.0	0.0	0.0	0.0	100.0	0.0	100.0
		6.00	0.0	0.0	0.0	0.0	0.0	100.0	100.0
Cross-validated	Count	1.00	79	0	1	0	0	0	80
		2.00	0	80	0	0	0	0	80
		3.00	0	0	80	0	0	0	80
		4.00	0	0	0	80	0	0	80
		5.00	0	0	0	0	80	0	80
		6.00	0	0	0	0	0	80	80
	%	1.00	98.8	0.0	1.3	0.0	0.0	0.0	100.0
		2.00	0.0	100.0	0.0	0.0	0.0	0.0	100.0
		3.00	0.0	0.0	100.0	0.0	0.0	0.0	100.0
		4.00	0.0	0.0	0.0	100.0	0.0	0.0	100.0
		5.00	0.0	0.0	0.0	0.0	100.0	0.0	100.0
		6.00	0.0	0.0	0.0	0.0	0.0	100.0	100.0

a 100.0% of original grouped cases correctly classified.

b 99.8% of cross-validated grouped cases correctly classified.

**Table 9.** Wilks' Lambda test for sensor selections.

**Modalitiy**	**Sensor Label**	**Wilks' Lambda**	**F**
E-NOSE	SENSOR 01	0.334	189.264
	SENSOR 02	0.465	109.242
	SENSOR 03	0.026	3,559.567
	SENSOR 04	0.064	1,384.462
	SENSOR 05	0.438	121.805
	SENSOR 06	0.036	2,549.234
	SENSOR 07	0.069	1,285.085
	SENSOR 08	0.214	348.496
	SENSOR 09	0.061	1,466.481
	SENSOR 10	0.066	1,333.454
	SENSOR 11	0.049	1,839.524
	SENSOR 12	0.174	448.954
	SENSOR 13	0.023	4,110.666
	SENSOR 14	0.739	33.483
	SENSOR 15	0.132	621.108
	SENSOR 16	0.307	214.050
	SENSOR 17	0.657	49.464
	SENSOR 18	0.309	212.426
	SENSOR 19	0.071	1,240.156
	SENSOR 20	0.007	13,299.374
	SENSOR 21	0.249	285.176
	SENSOR 22	0.128	645.636
	SENSOR 23	0.409	137.186
	SENSOR 24	0.073	1,201.120
	SENSOR 25	0.032	2,869.053
	SENSOR 26	0.048	1,896.044
	SENSOR 27	0.246	289.864
	SENSOR 28	0.348	177.933
	SENSOR 29	0.340	183.761
	SENSOR 30	0.025	3,694.859
	SENSOR 31	0.080	1,095.785
	SENSOR 32	0.168	468.469

ACOUSTIC	SENSOR 33	0.043	2,091.618
	SENSOR 34	0.066	1,335.055

**Table 10.** ANN classification results using LDA-CLNN.

**Method**	**Number of samples**		**Number of clusters detected % Correct Classification**	
	**Training and Cross Validation**	**Testing**		
**E-nose**	120 (25%)	* 360 (75%)	4	66.7%
**E-nose and acoustic**	120 (25%)	* 360 (75%)	6	84.4%

* None of the training and cross validation samples were used in the testing.
